# Supplementary material for: Hypertension among persons living with HIV—Zambia, 2021; A cross-sectional study of a national electronic health record system
Source: PLOS Glob Public Health. 2023 Jul 10;3(7):e0001686. doi: 10.1371/journal.pgph.0001686 (PMC10332593; doi:10.1371/journal.pgph.0001686)
Supplement: S1 File — Available from: https://www.croiconference.org/abstract/hypertension-prevalence-among-persons-living-with-hiv-zambia-july-2020-june-2021/. (PDF) [file pgph.0001686.s001.pdf]

# *Hypertension Prevalence among Persons Living with HIV – Zambia, July 2020–June 2021*

**Jonas Z. Hines[1],** Jose Tomas Prieto[2], Sombo Fwoloshi[3], Peter A. Minchella[1], Cecilia Chitambala[1], Dalila Zachary[1], Megumi Itoh[1], Khozya D. Zyambo[3], Lloyd B. Mulenga[3], Simon Agolory[1]
[1]Division of Global HIV and Tuberculosis, U.S. Centers for Disease Control and Prevention, Lusaka, Zambia; [2]Palantir Technologies, Paris, France; [3]Ministry of Health, Lusaka, Zambia

## BACKGROUND

Hypertension is a major risk factor for stroke and cardiovascular disease, both of which are common causes of death in Zambia. Data on hypertension prevalence in Zambia are scarce and generally limited to either specific populations or geographic areas. Persons living with HIV (PLHIV) are at increased risk of chronic diseases, so understanding prevalence and management of risk factors like hypertension are important. We sought to measure hypertension prevalence among PLHIV in Zambia using a national electronic health record (EHR).

## METHODS

- Retrospective cohort study of hypertension prevalence among PLHIV aged ≥18 years in Zambia
- Selected, de-identified demographic data, clinical information, and pharmacy records extracted from Zambia's SmartCare EHR system, which contains data on ~90% of PLHIV on treatment
- We analyzed data from PLHIV aged ≥18 years with at ≥2 active clinical visit from July 2020 to June 2021
- Data included demographics, past medical history, medications, blood pressure (BP) measurements, height and weight, and laboratory data (CD4+ count, HIV viral load, and creatinine). Variables for non-HIV medical history, diet, amount of physical activity, smoking history, cholesterol levels, and blood glucose were mostly missing
- Elevated BP defined as a systolic BP reading of ≥140 mmHg or diastolic BP readings of ≥90 mmHg.
- Hypertension defined as ≥2 elevated systolic or diastolic BP readings during the study period, or having an antihypertensive medication prescribed
- In PLHIV with hypertension, grade 2 hypertension defined as ≥1 systolic BP reading ≥160 mmHg or diastolic BP reading ≥100 mmHg; hypertensive urgency defined as ≥1 systolic BP reading ≥180 mmHg or diastolic BP reading ≥110 mmHg
- Multiple logistic regression utilized to assess associations between hypertension and independent variables with ≤10% missingness.

Among persons living with HIV in Zambia, **15.1%** had hypertension. Only 11.5% had an antihypertensive medication prescribed.

## RESULTS

- 133,206 (12.6%) of 1,056,556 PLHIV aged ≥18 years had ≥2 blood pressure readings (Figure)
- Mean age was 42.4 years (range 18-105 years) and 64.8% were females. The median time on ART was 6.0 years and 91.4% of PLHIV were on dolutegravir-based ART regimens
- 35.7% of PLHIV had ≥1 elevated BP reading
- Hypertension prevalence was 15.1% (95% CI: 14.9-15.3). Among PLHIV with hypertension, 62% had grade 2 hypertension and 27.9% had hypertensive urgency.
- Just 11.5% PLHIV with hypertension had an anti-hypertensive medication recorded in their EHR in the past five years, and of those, 64.6% still had an elevated BP
- The odds of hypertension were greater in males, advanced age groups, urban areas, and overweight and obese PLHIV (Table)

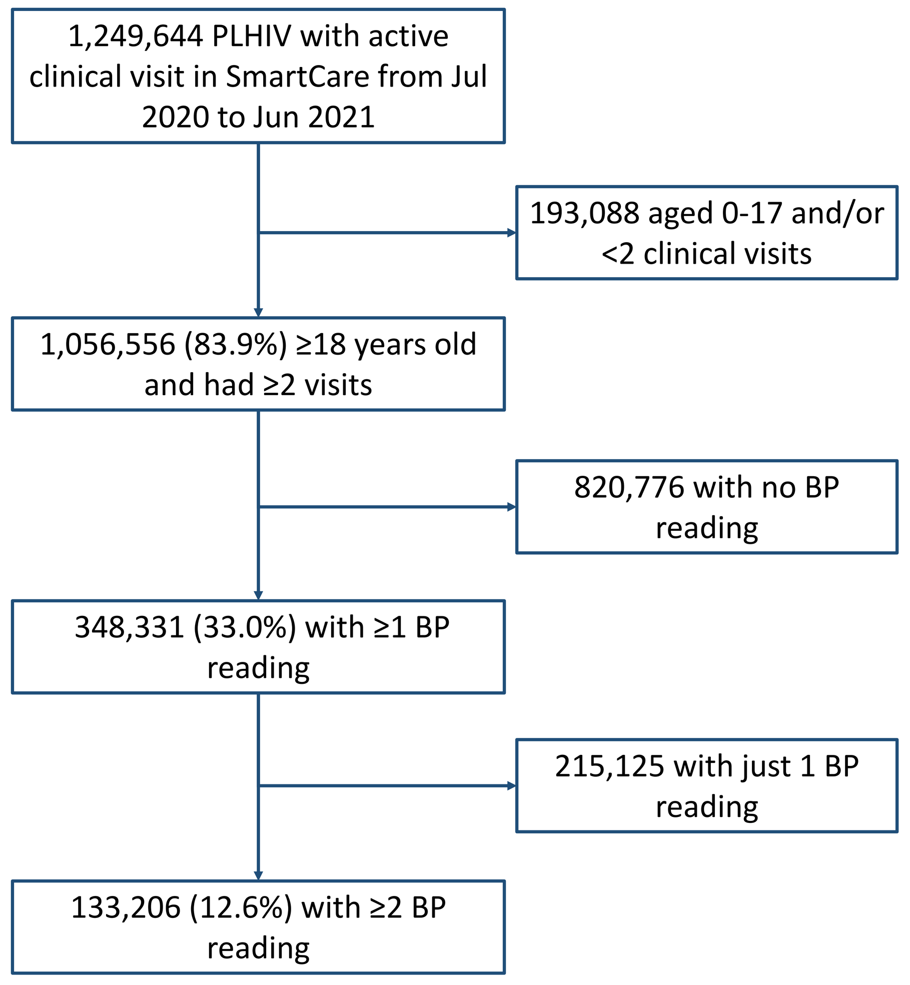


*Figure. Flow diagram of hypertension prevalence study among persons living with HIV in Zambia*

## CONCLUSIONS

- Hypertension was common among a cohort of PLHIV in Zambia
- Many PLHIV with hypertension had dangerously high blood pressure and few had documentation of being on antihypertensive treatment
- Interventions to strengthen integrated management of non-communicable diseases in clinics providing ART might help to increase diagnosis and treatment of hypertension in Zambia
- Data completeness for key variables in the EHR including BP readings was very low
- Training on the importance of accurate and complete data capture at the point-of-service might help improve surveillance of hypertension (and other non-communicable diseases) in Zambia

*Table. Hypertension prevalence and odds ratios by patient characteristics among persons living with HIV (N=133,206) — Zambia, July 2020 – June 2021*

| | Prevalence (N=133,206) | Odds ratio (95% CI) | Adjusted OR (95% CI) † |
|---|---|---|---|
| Overall | 15.1 | - | - |
| Sex | | | |
| Female | 14.0 | Referent | |
| Male | 17.2 | 1.28 (1.24, 1.32) | 1.30 (1.25, 1.35) |
| Age group | | | |
| 18-29 | 3.9 | Referent | |
| 30-44 | 10.0 | 2.72 (2.52, 2.95) | 2.48 (2.25, 2.72) |
| 45-59 | 21.5 | 6.67 (6.17, 7.20) | 5.74 (5.22, 6.31) |
| ≥60 | 37.6 | 14.72 (13.53, 16.02) | 13.90 (12.53, 15.43) |
| Urban/rural designation | | | |
| Rural | 11.1 | Referent | Referent |
| Urban | 16.8 | 1.62 (1.56, 1.68) | 1.44 (1.38, 1.50) |
| Years on ART | | | |
| 0-1 | 11.4 | Referent | Referent |
| 2-4 | 13.1 | 1.18 (1.11, 1.24) | 0.88 (0.82, 0.95) |
| 5-9 | 14.9 | 1.36 (1.29, 1.44) | 0.82 (0.76, 0.88) |
| ≥10 | 20.1 | 1.96 (1.85, 2.06) | 0.9 (0.83, 0.97) |
| ART regimen‡ | | | |
| Efavirenz-based | 12.3 | Referent | Referent |
| Dolutegravir-based | 15.3 | 1.28 (1.19, 1.39) | 1.08 (0.98, 1.19) |
| Other | 14.3 | 1.18 (1.06, 1.32) | 0.99 (0.86, 1.13) |
| Script length | | | |
| <3 months | 12.9 | Referent | Referent |
| 3-5 months | 14.7 | 1.16 (1.08, 1.24) | 1 (0.91, 1.1) |
| ≥6 months | 15.5 | 1.24 (1.16, 1.33) | 1.02 (0.93, 1.12) |
| Body mass index | | | |
| Low (<18.5) | 8.0 | 0.66 (0.61, 0.70) | 0.62 (0.58, 0.67) |
| Normal (18.5-24.9) | 11.6 | Referent | Referent |
| Overweight (25-29.9) | 21.6 | 2.09 (2.01, 2.17) | 1.91 (1.83, 1.99) |
| Obese (≥30.0) | 29.9 | 3.24 (3.11, 3.39) | 2.92 (2.78, 3.08) |
| Baseline CD4+ count | | | |
| 0-200 | 16.3 | Referent | |
| 201-350 | 17.1 | 1.06 (0.98, 1.14) | NC |
| >350 | 16.4 | 1.01 (0.95, 1.08) | NC |
| Most recent CD4+ count‡ | | | |
| 0-200 | 16.1 | Referent | |
| 201-350 | 17.3 | 1.08 (1.01, 1.16) | NC |
| >350 | 16.3 | 1.01 (0.95, 1.07) | NC |
| Most recent viral load count¶ | | | |
| <1000 | 15.5 | Referent | Referent |
| 1,000-9,999 | 12.2 | 0.76 (0.67, 0.86) | 1.04 (0.89, 1.2) |
| ≥10,000 | 9.9 | 0.60 (0.53, 0.67) | 0.92 (0.80, 1.06) |
| Renal function** | | | |
| Normal creatinine | 18.0 | Referent | |
| Elevated creatinine | 30.2 | 1.97 (1.79, 2.18) | NC |

* Hypertension defined as ≥2 systolic blood pressure readings of ≥140 mmHg or ≥2 diastolic blood pressure readings of ≥90 mmHg during the study period
† Only variables with <10% missingness included in multivariable logistic regression
‡ The most recently listed ART regimen in SmartCare. If a regimen listed both dolutegravir and efavirenz, then it was excluded from the analysis (n = 377)
¶ Conducted during the study period (i.e., Jul 2020-Jun 2021)
** Elevated creatinine defined as ≥115 µmol/L in men and ≥98 µmol/L in women
CI: confidence interval; NC: not calculated; OR: odds ratio

## ADDITIONAL INFORMATION

This work has been supported by the Zambia Ministry of Health and the U.S. President's Emergency Plan for AIDS Relief (PEPFAR) through the Centers for Disease Control and Prevention. The findings and conclusions in this report are those of the authors and do not necessarily represent the official position of the Centers for Disease Control and Prevention

Author contact: Jonas Z. Hines (jhines1@cdc.gov)
