# Supplementary material for: Hypertension among persons living with HIV—Zambia, 2021; A cross-sectional study of a national electronic health record system
Source: PLOS Glob Public Health. 2023 Jul 10;3(7):e0001686. doi: 10.1371/journal.pgph.0001686 (PMC10332593; doi:10.1371/journal.pgph.0001686)
Supplement: Attachment — Submitted filename: PGPH-D-23-00227 response to reviewers comments.docx [file pgph.0001686.s003.docx]

4 April 2023

Dear PLoS Global Public Health editor,

Thank you for the opportunity to revise our manuscript, “Hypertension among persons living with HIV — Zambia, 2021; A cross-sectional study of a national electronic health record system,” in PLoS Global Public Health journal. We are pleased to submit our revised manuscript responding to the editors and reviewers’ comments. We have attempted to respond as best as we can by updating the manuscript and expanding the appendix. Below you will find specific responses to each of the comments.

Additionally, we wish to draw your attention to four coauthors who were mistakenly omitted from the initial submission to PLoS GPH. This mistake was a version control issue related to the internal CDC clearance process. All these authors met ICMJE criteria for coauthorship but submitted their author concurrence statement after entry into the CDC clearance portal and the subsequent cleared version was what was entered into PLoS GPH. Please accept our apologies in this omission and let us know if you have any concerns about their addition at this stage.

Thank you for taking the time to review our re-submission. We hope you agree the changes sufficiently address the remaining items. We look forward to your response.

Sincerely,
Jonas Hines

PGPH-D-23-00227
Hypertension among persons living with HIV — Zambia, 2021; A cross-sectional study of a national electronic health record system
PLOS Global Public Health

Dear Dr. Hines,

Thank you for submitting your manuscript to PLOS Global Public Health. After careful consideration, we feel that it has merit but does not fully meet PLOS Global Public Health’s publication criteria as it currently stands. Therefore, we invite you to submit a revised version of the manuscript that addresses the points raised during the review process.

You have observed that your findings in the multivariable model suggest the increased odds of hypertension among older age groups in PLHIV. This intuitive finding is similar to what is commonly observed in the general population. While this is an important observation, your study could benefit from further analysis, utilizing the advantage of a large sample size. For example, are there any prognostic variables associated with higher hypertension apart from age, specifically in PLHIV? Additional analyses to identify and present specific attributes related to hypertension in PLHIV could provide additional insights into the complex relationship between HIV and hypertension. Also, please expand the discussion section of your manuscript to include a comparison of your study findings with the general population (non-PLHIV). This comparison should highlight the similarities and contrasts between findings in PLHIV and the general population and provide a broader context for understanding the implications of the results.
*Author response*: In this analysis, we evaluated variables for which there was sufficient data completeness, including only variables with a pre-specified ≥90% completeness threshold in the multivariable model. Variables analyzed included sex, age, province, urban/rural designation, years on ART, ART regimen, ART prescription length, body mass index (BMI), CD4+ count, HIV viral load, and creatinine. Only age, urban/rural designation, and ART prescription length were significant factors associated with hypertension in the multivariable model. We have strengthened the discursive language in the discussion on these findings. Additionally, BMI and creatinine did not meet pre-specified completeness thresholds to include the multivariable model, although we did include results and discussion about their associations from the bivariable analysis given their potential interest. Unfortunately, information on other potentially interesting factors like opportunistic infection history, non-HIV past medical history, other cardiovascular risk factors, and/or socioeconomic status was not available from this data source. Lastly, we have added language to the discussion comparing our findings with hypertension among the general population.

Please submit your revised manuscript by Apr 19 2023 11:59PM. If you will need more time than this to complete your revisions, please reply to this message or contact the journal office at [globalpubhealth@plos.org](mailto:globalpubhealth@plos.org). When you're ready to submit your revision, log on to <https://www.editorialmanager.com/pgph/> and select the 'Submissions Needing Revision' folder to locate your manuscript file.

Please include the following items when submitting your revised manuscript:

- A rebuttal letter that responds to each point raised by the editor and reviewer(s). You should upload this letter as a separate file labeled 'Response to Reviewers'.
- A marked-up copy of your manuscript that highlights changes made to the original version. You should upload this as a separate file labeled 'Revised Manuscript with Track Changes'.
- An unmarked version of your revised paper without tracked changes. You should upload this as a separate file labeled 'Manuscript'.

Guidelines for resubmitting your figure files are available below the reviewer comments at the end of this letter.

We look forward to receiving your revised manuscript.

Kind regards,

Giridhara R Babu, MBBS, MPH, PhD
Academic Editor
PLOS Global Public Health

Journal Requirements:

1. Please review your reference list to ensure that it is complete and correct. If you have cited papers that have been retracted, please include the rationale for doing so in the manuscript text, or remove these references and replace them with relevant current references. Any changes to the reference list should be mentioned in the rebuttal letter that accompanies your revised manuscript. If you need to cite a retracted article, indicate the article’s retracted status in the References list and also include a citation and full reference for the retraction notice.

*Author response*: We have reviewed the reference list.

2. Please include the following request in the decision letter, and ping me with follow up. “Please include a complete copy of PLOS’ questionnaire on inclusivity in global research in your revised manuscript. Our policy for research in this area aims to improve transparency in the reporting of research performed outside of researchers’ own country or community. The policy applies to researchers who have travelled to a different country to conduct research, research with Indigenous populations or their lands, and research on cultural artefacts. The questionnaire can also be requested at the journal’s discretion for any other submissions, even if these conditions are not met.  Please find more information on the policy and a link to download a blank copy of the questionnaire here: <https://journals.plos.org/plosone/s/best-practices-in-research-reporting>. Please upload a completed version of your questionnaire as Supporting Information when you resubmit your manuscript.”

*Author response*: We have completed the questionnaire and included it with our revised submission.

3. Please amend your detailed Financial Disclosure statement. This is published with the article. It must therefore be completed in full sentences and contain the exact wording you wish to be published.

a. State the initials, alongside each funding source, of each author to receive each grant.
b. State what role the funders took in the study. If the funders had no role in your study, please state: “The funders had no role in study design, data collection and analysis, decision to publish, or preparation of the manuscript.”
c. If any authors received a salary from any of your funders, please state which authors and which funders.

If you did not receive any funding for this study, please simply state: “The authors received no specific funding for this work.”

*Author response*: We have added the phrase, “The authors received no specific funding for this work” to the funding acknowledgement statement. We are paid employees of our respective institutions but this project did not receive specific fundings.

4. Please provide separate figure files in .tif or .eps format only and remove any figures embedded in your manuscript file. Please also ensure that all files are under our size limit of 10MB.

For more information about figure files please see our guidelines:

<https://journals.plos.org/globalpublichealth/s/figures> 
<https://journals.plos.org/globalpublichealth/s/figures#loc-file-requirement>

*Author response*: We have updated the figure (flow diagram) to be .tif and included in our revised submission.

5. We have noticed that you have uploaded Supporting Information files, but you have not included a list of legends. Please add a full list of legends for your Supporting Information files after the references list.

*Author response*: We have added this section to the manuscript file, indicating this is a poster presented at CROI conference on the same topic.

Additional Editor Comments (if provided):


[Note: HTML markup is below. Please do not edit.]

Reviewers' comments:

Reviewer's Responses to Questions

**Comments to the Author**

1. Does this manuscript meet PLOS Global Public Health’s [publication criteria](https://journals.plos.org/globalpublichealth/s/criteria-for-publication)? Is the manuscript technically sound, and do the data support the conclusions? The manuscript must describe methodologically and ethically rigorous research with conclusions that are appropriately drawn based on the data presented.

Reviewer #1: Partly

Reviewer #2: Partly

Reviewer #3: Yes

2. Has the statistical analysis been performed appropriately and rigorously?

Reviewer #1: Yes

Reviewer #2: Yes

Reviewer #3: Yes

3. Have the authors made all data underlying the findings in their manuscript fully available (please refer to the Data Availability Statement at the start of the manuscript PDF file)?

The [PLOS Data policy](https://journals.plos.org/globalpublichealth/s/data-availability) requires authors to make all data underlying the findings described in their manuscript fully available without restriction, with rare exception. The data should be provided as part of the manuscript or its supporting information, or deposited to a public repository. For example, in addition to summary statistics, the data points behind means, medians and variance measures should be available. If there are restrictions on publicly sharing data—e.g. participant privacy or use of data from a third party—those must be specified.

Reviewer #1: No

Reviewer #2: Yes

Reviewer #3: Yes

4. Is the manuscript presented in an intelligible fashion and written in standard English?

PLOS Global Public Health does not copyedit accepted manuscripts, so the language in submitted articles must be clear, correct, and unambiguous. Any typographical or grammatical errors should be corrected at revision, so please note any specific errors here.

Reviewer #1: Yes

Reviewer #2: Yes

Reviewer #3: Yes

5. Review Comments to the Author

Please use the space provided to explain your answers to the questions above. You may also include additional comments for the author, including concerns about dual publication, research ethics, or publication ethics. (Please upload your review as an attachment if it exceeds 20,000 characters)

Reviewer #1: This is an insightful study that will enable strengthening of health service delivery to PLHIV and other health system components such as Health Information System: Data entry, storage and capacity building among health care workers.

A number of issues to consider:

1) The use of the term 'common' in the first sentence in the discussion both in the abstract and discussion section (main body) could suggest high prevalence. The study findings show a prevalence of about 15%, compared to the prevalence rates of hypertension in Africa (16%), the global rates among PLHIV (20-25%) and Zambian DHS (8.8%) as presented in the introduction section. A comparator could complement the statement and increase clarity. Also, do we have statistics on hypertension among PLHIV in Africa or Southern Africa region, inclusion of the same, could help show the magnitude of the hypertension among the population besides the general population statistics.

*Author response*: We have update language in the discussion to add more description to our statement in response to this comment (“Hypertension was common among PLHIV in Zambia, with one in about every seven PLHIV aged ≥18 years having hypertension during a one-year period. This estimate is similar to other studies of PLHIV in sub-Saharan Africa, if not slightly lower [12,30].”)

2) STUDY OBJECTIVE: The objective stated in line 83-85 should collate to the methodology and other parts of the results. As presented in lines 134-145, the study also aimed to assess associated factors to the hypertension in PLHIV besides measuring the proportion of hypertension. As stated in the background section it only indicates the study as a prevalence study.

*Author response*: We have added “and associated factors” to the objectives sentence as suggested by the reviewer.

3) Clarification on hypertension case definition in line 127. The hypertension diagnosis requires 'at least' 2 readings.

*Author response*: This is correct, at least two readings. We have left the symbol “≥” in the manuscript but if the editors wish us to replace with “at least,” we are happy to update the language here and elsewhere.

4) Use of the term 'many' in line 189

*Author response*: We believe this comment refers to the word ‘many’ not being specific enough to refer to the statistic that 60.7% of PLHIV with hypertension had grade 2 hypertension and 27.0% had hypertensive urgency. We have updated the sentence to be more specific (“Most PLHIV with hypertension had dangerously high blood pressure readings (i.e., grade 2 hypertension) putting them at elevated risk for cardiovascular disease including CVAs and acute cardiovascular events”).

5) Line 208 'high sodium diet'. Consider rephrasing e.g., consumption of diets high in sodium or consumption of unhealth diets such as diets high in sodium to improve clarity on the plausible exposing factors, since there are other dietary factors that also predispose the population to hypertension or cardiovascular risk factors.

*Author response*: We incorporated this comment and updated the sentence to read, “The observed geographic patterns of hypertension could be related to regional urban/rural differences in diet environmental factors associated with hypertension (i.e., consumption of unhealthy diets such as diets high in sodium urban dwelling PLHIV consuming higher sodium diets, greater exposure to pollution exposure than those in rural areas, or reduced opportunities for physical activity) and/or be an artifact of poor data quality (i.e., highest proportion of PLHIV with hypertension observed in provinces that also had better data completeness).”

6) Study limitation; consider adding a limitation on how most of the data is from urban facilities (77%) and from Lusaka and Southern provinces (79%) limits the interpretation of the results.

*Author response*: We have updated the limitations section to include the sentence, “Furthermore, the data for this analysis are mostly from urban facilities in Lusaka and Southern Provinces.”

7) The article could also highlight the implication of data availability. As presented in table 1, most the data is from urban facilities, and how it could be important to strengthen the health information system to support health service delivery and practice (health care).
*Author response*: We have added a sentence to the discussion of EHRs that reads, “This analysis suggests EHRs are being more widely implemented and adopted in urban areas, which has the potential to affect urban-rural differences in surveillance and/or care.”

8) Capitalise 'all' in line 285

*Author response*: This typo has been fixed. Thank you.

Reviewer #2: Thank you for this scientific work which reflects the data gaps in electronic surveillance systems in developing countries. However, please note in the Method: the TYPE OF CROSS-SECTIONAL STUDY (DESCRIPTIVE OR ANALYTICAL) you have carried out; the type of sample and the sampling technique used. In addition, data such as the mean, median and measure of variance are not available in the tables.

*Author response*: The methods section contains the following sentence: “We conducted a cross-sectional study of hypertension among PLHIV aged ≥18 years in Zambia from January to December 2021 (the last full calendar year of data available)” and the analytic methods are described on lines 127-146. There was no sampling technique for this secondary analysis or routinely collected health information but the inclusion criteria are stated on lines 111-113 (“Only PLHIV aged ≥18 years with ≥2 clinical visits in 2021 in SmartCare EHR were eligible for inclusion in the study (in Zambia, BP is measured at each clinical visit but not necessarily at other patient interactions captured in SmartCare EHR [e.g., lab check, pharmacy pick-up])”). Lastly, we report proportion and 95% confidence intervals (CIs) in the tables but omitted from the body of the paper to declutter the text. This is a stylistic decision, and we defer to the editors if 95% CIs should be added into the text of the paper.

Reviewer #3: Very well conducted. Comprehensive research study.

Only addition can be if researchers can highlight on any peculiarity in PLHIV cohort as compared to general population in trend of hypertension otherwise age bracket, gender, urban-rural divide are giving the same trend as of general population.

*Author response*: Please see response above. The findings from this analysis demonstrate more similarities than differences with the general population (although data are limited for the latter in Zambia).
